# Warning indicators of COVID-19 severity: a retrospective observational study integrating modern biomarkers and traditional tongue features

**DOI:** 10.3389/fmed.2025.1500605

**Published:** 2025-04-15

**Authors:** Zhang Jing, Liu Yuntao, Zheng Danwen, Ye Gangfu, Chen Qiumin, Huang Jianshan, Wang Jiamei, Ma Zengming, Zhang Zhongde

**Affiliations:** ^1^Department of Pulmonary and Critical Care Medicine, Xiamen Hospital of Traditional Chinese Medicine, Xiamen, Fujian, China; ^2^Guangzhou University of Chinese Medicine, Guangzhou, Guangdong, China; ^3^Key Laboratory of Infectious Diseases, Guangdong Provincial Bureau of Chinese Medicine, Guangzhou, China; ^4^Emergency Department, The Second Affiliated Hospital of Guangzhou University of Chinese Medicine, Guangzhou, Guangdong, China; ^5^Department of Traditional Chinese Medicine, Xinglin Branch of the First Affiliated Hospital of Xiamen University, Xiamen, Fujian, China; ^6^Department of Pediatrics, Xiamen Hospital of Traditional Chinese Medicine, Xiamen, Fujian, China; ^7^Department of Traditional Chinese Medicine, Xiamen Fifth Hospital, Xiamen, Fujian, China; ^8^Integrated TCM & Western Medicine Department, Xiamen Xianyue Hospital, Xiamen, Fujian, China

**Keywords:** COVID-19, severity prediction, traditional Chinese medicine, tongue diagnosis, biomarkers

## Abstract

**Objective:**

This study aims to identify early warning indicators of COVID-19 severity by integrating modern medical biomarkers with traditional Chinese medicine (TCM) tongue features.

**Methods:**

A retrospective observational study was conducted on 409 hospitalized COVID-19 patients from two centers in China. Patients were stratified into severe (*n* = 50) and non-severe (*n* = 359) groups based on the 10th edition of China’s diagnostic guidelines. Data included demographics, clinical symptoms, tongue characteristics, and laboratory parameters. Univariate analyses (chi-square/Fisher’s exact tests) and stepwise logistic regression were performed to identify key predictors.

**Results:**

Age (*p* < 0.001), fever (*p* < 0.001), elevated procalcitonin (PCT, *p* < 0.001), thick tongue fur (*p* = 0.003), and fat tongue shape (*p* = 0.002) were significant predictors of severity. The combined model integrating these factors demonstrated superior predictive performance (Nagelkerke *R*^2^ = 0.741).

**Conclusion:**

Integrating TCM tongue features (thick fur and fat shape) with clinical biomarkers (age, fever, and PCT) enhances early identification of severe COVID-19, particularly in resource-limited settings.

## 1 Introduction

As of 12 July 2024, the World Health Organization (WHO) (1) reports that the global pandemic of novel Coronavirus Disease 2019 (COVID-19) has resulted in over 700 million confirmed cases and 7 million deaths. History teaches us that the next pandemic is a matter of “when,” not “if” (2). Strategies for preventing severe outcomes and improving treatment efficacy remain critical areas requiring ongoing and relentless research in COVID-19 management. In this study, we focus on obtaining warning indicators of severity by using the retrospective observational method.

To date, numerous studies have explored warning indicators for COVID-19. These include baseline characteristics, such as age, diabetes, obesity, hypertension, and genetic risk (3–7), clinical symptoms, such as chest pain, dyspnea, and headache (8), and laboratory markers, such as procalcitonin (PCT), D-dimer (D-D), lactic dehydrogenase (LDH), c-reactive protein (CRP), lymphocyte (LY), tumor necrosis factor-*α* (TNF-α), and serum cystatin C (sCys C) (9–13). However, the majority of existing research relies on cross-sectional or retrospective designs, lacking comprehensive integration of multidisciplinary approaches.

Traditional Chinese medicine (TCM), akin to other global traditional medical systems, posits that external manifestations (e.g., tongue features) can predict disease progression. For instance, tongue diagnosis has been shown to aid in predicting acute heart failure, classifying diabetes and hypertension, and identifying diagnostic signatures in coronary artery disease with clopidogrel resistance (14–17). These non-invasive diagnostic methods are particularly valuable in resource-limited settings where advanced testing equipment is unavailable. Despite this potential, research on TCM-based warning indicators for COVID-19 severity remains scarce.

In this study, we conducted a retrospective observational analysis combining modern medical indicators (e.g., laboratory markers) and TCM tongue features. These variables were analyzed using one-way ANOVA and logistic regression to identify severity-related warning indicators. This approach aims to bridge the gap between modern and traditional medicine, offering a supplementary tool for early risk stratification in diverse clinical settings.

## 2 Materials and methods

### 2.1 Cases

A total of 409 cases of novel coronavirus infection were admitted to sentinel hospitals in Guangzhou, Guangdong, and Xiamen, Fujian, China, from May to October 2021.

### 2.2 Inclusion and exclusion criteria

According to China’s 10th edition of the Diagnosis and Treatment Guidelines for Novel Coronavirus Infection (Trial) (18), a positive nucleic acid test for the novel coronavirus was the diagnostic criterion. During this period, all patients were infected with the Delta strain, and cases with psychiatric conditions were excluded. The diagnostic criteria for severity are listed in Table 1. A diagnosis could be made if any one of the following criteria was met. Cases meeting these criteria were included in the severe group, while others were placed in the non-severe group.

**Table 1:** Diagnostic criteria for severity.

<14 y	14 y~
High fever >3 days;	Respiratory rate ≥ 30 breaths/min;
Increased respiratory rate in non-fever and emotionally affected conditions: <2 m, RR ≥ 60 breaths/min; 2–12 m, RR ≥ 50; 1–5 y, RR ≥ 40; >5 y, RR ≥ 30	Resting-state without oxygen, SpO_2_ ≤ 93%;
Resting-state without oxygen, SpO_2_ ≤ 93%	Oxygenation index ≤300 mmHg;
Nasal flaring, triple concave sign, wheezing, or stridor	Clinical symptoms aggravated, and imaging progression of more than 50% in 24–48 h.
Impaired consciousness or convulsions	
Refusal to eat or difficulty in feeding, signs of dehydration.	

All ages
Respiratory failure requires mechanical ventilation.
Shock
Other organ failure required intensive care unit (ICU) treatment.

### 2.3 Data collection

Data were collected by clinicians and cross-verified by two independent researchers. Inaccurate data were discarded and treated as missing. Abbreviations are provided in Appendix Table 1.

General information includes gender, age, body mass index (BMI), heavy smoking, hypertension, heart disease, diabetes, chronic lung disease, chronic liver disease, chronic kidney disease, neoplasms, rheumatologic and immunologic diseases, surgery, and vaccination history, totaling 15 items.

Clinical symptoms include fever, cold, headache, sweating, nasal congestion and runny nose, cough, sputum, sore throat, shortness of breath, chest tightness, nausea and vomiting, fatigue, loss of taste or smell, diarrhea, muscular pains, and poor appetite, totaling 18 items. Data for the severe group were collected 4 days before the diagnosis of severity, while data for the non-severe group were collected between 7 and 11 days of disease duration. The aim of the study was to detect critically ill patients as early as possible. Therefore, data from 4 days before the diagnosis of severity were selected for the severe group. In contrast, the non-severe group was pre-measured and found to have worse data between days 7 and 11 of the disease course; hence, this period was selected for comparison.

Tongue features were evaluated according to the tongue assessment criteria (19), with two senior staff members providing their evaluations after careful deliberation. The classification of tongue features is detailed in Table 2. Examples of assessments are provided in Appendix Table 2.

**Table 2:** Tongue classification (29 items).

Classification	Content
Tongue color	Pale white, light red, red, reddish-red, purple, blue, bruise;
Tongue shape	Withered, old, tender, fat, thin, punctured, cracked, teeth marks;
Color of tongue fur	White, yellow, gray, black;
Quality of tongue fur	Scanty, thick, moist, slippery, dry, greasy, rotten, peeling, like peeling.

Testing information was categorized as normal or abnormal based on the reference range provided by the test results. The criteria for judgment are provided in Appendix Table 3. The Tongue classification items are presented in Table 3. As the study objective was to identify early warning indicators rather than precise disease manifestations, abnormal laboratory findings were not subcategorized into elevated or decreased levels. Comprehensive data characterization, including median values and interquartile ranges (IQR), are provided in Appendix Table 4. Data for the severe group were collected 4 days before the diagnosis of severity, while data for the non-severe group were collected between 7 and 11 days of disease duration.

**Table 3:** Laboratory markers (22 items).

Classification	Items
Blood counts	White blood cell (WBC), neutrophil (NE), LY, hemoglobin (HGB), platelet (PLT);
Inflammatory indicators	CRP, interleukin 6 (IL-6), PCT;
Coagulation tests	Activated partial thromboplastin time (APTT), prothrombin time (PT), thrombin time (TT), fibrinogen (Fib), fibrinogen degradation products (FDP), D-D, international normalized ratio (INR), prothrombin activity (PTA);
Liver and kidney routine	Creatine kinase (CK), creatine kinase isoenzyme (CKMB), glutamic-pyruvic transaminase (ALT), glutamic oxaloacetic transaminase (AST), creatinine (Cr), LDH.

### 2.4 Statistical analysis methods

Excel was used for data entry, and SPSS Statistics for Windows, version 18.0 (SPSS Inc., Chicago, Ill., USA) was used for statistical analysis. Continuous variables were checked for maximum and minimum values, and histograms and quartiles were used to examine the data. Categorical variables were assessed using frequency tables, which were sorted to screen for and exclude erroneous data. Measured data were described using the mean ± standard deviation for conformity to a normal distribution, otherwise median and interquartile spacing median were used (P25, P75). Frequency and percentage were used to express the count data. General information, clinical symptoms, tongue features, and testing information were categorized as count data. Univariate analysis of the count data between groups was performed using the chi-square test or Fisher’s exact probability test. Frequency indicators with a severe positive rate of <3% and secondary indicators with obvious correlations were discarded based on medical and testing principles. The resulting positive indicators were analyzed using binary logistic regression. A *p* < 0.05 was considered statistically significant.

## 3 Results

A total of 409 cases with novel coronavirus infection were included: 50 cases in the severe group and 359 cases in the non-severe group.

### 3.1 Univariate analysis

In the univariate analysis of general information, indicators with a positive rate of <3% for severity (such as heavy smoking, heart disease, diabetes, chronic lung disease, chronic liver disease, chronic kidney disease, tumors, rheumatologic and immunologic diseases, and surgery) were discarded. Using *p* < 0.05 as the inclusion criterion, four indicators, namely age (*p* < 0.001), BMI (*p* = 0.036), hypertension (*p* < 0.001), and vaccination (*p* < 0.001), were included, as shown in Table 4.

**Table 4:** Univariate analysis of general information.

General information	*n*	Category	Non-severe group		Severe group		Positivity rate for severity		*χ^2^*	*P*
			*n*	%	*n*	%	*n*	%		
Sex	409	Female	195	88.64	25	11.36	50	100.00	0.329	0.566
		Male	164	86.77	25	13.23				
Age	409	<14	46	100.00	0	0.00	50	100.00	71.944	<0.001^#,a^
		14~	183	96.32	7	3.68				
		45~	97	82.91	20	17.09				
		65~	28	65.12	15	34.88				
		80~	5	38.46	8	61.54				
BMI	322	<18.5	44	100.00	0	0.00	27	54.00		0.017^*,a^
		18.5~	163	92.61	13	7.39				
		24~	69	87.34	10	12.66				
		28~	19	82.61	4	17.39				
Heavy smoking	409	N	354	88.06	48	11.94	2	0.49		0.206^a^
		Y	5	71.43	2	28.57				
Hypertension	409	N	321	90.93	32	9.07	18	4.40	23.989	<0.001^#^
		Y	38	67.86	18	32.14				
Heart disease	409	N	354	88.94	44	11.06	6	1.47	15.032	<0.001^#^
		Y	5	45.45	6	54.55				
Diabetes	409	N	347	89.43	41	10.57	9	2.20	16.465	<0.001^#^
		Y	12	57.14	9	42.86				
Chronic lung disease	409	N	335	88.16	45	11.84	5	1.22	0.315	0.547
		Y	24	82.76	5	17.24				
Chronic liver disease	409	N	356	87.68	50	12.32	0	0.00		1.000^a^
		Y	3	100.00	0	0.00				
Chronic kidney disease	409	N	357	88.15	48	11.85	2	0.49		0.075^a^
		Y	2	50.00	2	50.00				
Neoplasms	409	N	355	88.09	48	11.91	2	0.49		0.159^a^
		Y	4	66.67	2	33.33				
rheumatologic and immunologic diseases	409	N	357	87.71	50	12.29	0	0.00		1.000^a^
		Y	2	100.00	0	0.00				
Surgery	409	N	331	88.50	43	11.50	7	1.71	1.437	0.231
		Y	28	80.00	7	20.00				
Vaccination history	397	N	124	78.48	34	21.52	14	3.53	21.950	<0.001^#^
		Y	225	94.14	14	5.86				

N, No; Y, Yes. ^a^Fisher’s exact probability method, ^*^P < 0.05, ^**^P < 0.01, ^#^*P* < 0.001.

In the univariate analysis of clinical symptoms, indicators with a positive rate of <3% for severity (such as cold, headache, sweating, nasal congestion and runny nose, sore throat, chest tightness, nausea and vomiting, loss of taste or smell, diarrhea, and muscle pain) were discarded. Finally, six indicators, namely fever (*p* < 0.001), cough (*p* < 0.001), sputum (*p* < 0.001), shortness of breath (*p* < 0.001), fatigue (*p* < 0.001), and poor appetite (*p* < 0.001), were included, as shown in Table 5.

**Table 5:** Univariate analysis of clinical symptoms.

Symptom *n* = 409		Non-severe group		Severe group		Positivity rate for severity		*χ^2^*	*P*
		*n*	%	*n*	%	*n*	%		
Fever	N	277	97.9	6	2.1	44	10.76	87.413	<0.001^#^
	Y	82	65.1	44	34.9				
Cold	N	357	87.9	49	12.1	1	0.24		0.324^a^
	Y	2	58.8	1	41.2				
Headache	N	349	89.0	43	11.0	7	1.71	11.183	<0.001^#^
	Y	10	88.24	7	11.76				
Sweating	N	358	87.7	50	12.3	0	0.00		0.878^a^
	Y	1	100.0	0	0.0				
Nasal congestion and runny nose	N	321	86.8	49	13.2	1	0.24	2.821	0.093
	Y	38	97.4	1	2.6				
Cough	N	152	94.4	9	5.6	41	10.02	10.893	<0.001^#^
	Y	207	83.5	41	16.5				
Sputum	N	225	93.4	16	6.6	34	8.31	17.061	<0.001^#^
	Y	134	79.8	34	20.2				
Sore throat	N	330	87.5	47	12.5	3	0.73	0.054	0.817
	Y	29	90.6	3	9.4				
Shortness of breath	N	345	92.5	28	7.5	22	5.38	82.992	<0.001^#^
	Y	14	38.9	22	61.1				
Chest tightness	N	350	88.8	44	11.2	6	1.47	8.669	0.003^**^
	Y	9	60.0	6	40.0				
Nausea and vomiting	N	346	88.0	47	12.0	3	0.73	0.179	0.692
	Y	13	81.3	3	18.7				
Fatigue	N	333	90.0	37	10.0	13	3.18	15.793	<0.001^#^
	Y	26	66.7	13	33.3				
Loss of taste or smell	N	350	87.5	50	12.5	0	0.00	0.381	0.537
	Y	9	100.0	0	0.0				
Diarrhea	N	348	88.3	46	11.7	4	0.98	1.791	0.181
	Y	11	73.3	4	26.7				
Muscle pains	N	342	88.1	46	11.9	4	0.98	0.407	0.523
	Y	17	81.0	4	19.0				
Poor appetite	N	328	92.1	28	7.9	22	5.38	48.664	<0.001^#^
	Y	31	58.5	22	41.5				

N, No; Y, Yes. ^a^Fisher’s exact probability method, ^*^P < 0.05, ^**^P < 0.01, ^#^*P* < 0.001.

In the univariate analysis of tongue features, indicators with a positive rate of <3% for severity (such as pale white, pale red, red, reddish-red, blue, bruise, withered, old, tender, fat, thin, punctured, cracked, white, gray, black, weak, moist, dry, rotten, peeling, or like peeling) were excluded. Finally, five indicators, namely purple (*p* < 0.001), fat (*p* < 0.001), teeth marks (*p* = 0.04), thick (*p* < 0.001), and greasy (*p* = 0.031), were included, as shown in Table 6.

**Table 6:** Univariate analysis of tongue features.

Features *n* = 276		Non-severe group		Severe group		Positivity rate for severity		*χ^2^*	*P*
		*n*	%	*n*	%	*n*	%		
Tongue color									
Pale white	N	243	92.40	20	7.60	0	0.00		0.608^a^
	Y	13	100.00	0	0.00				
Light red	N	162	90.50	17	9.50	3	1.09	3.839	0.050
	Y	94	96.91	3	3.09				
Red	N	203	92.27	17	7.73	3	1.09	0.101	0.747
	Y	53	94.64	3	5.36				
Reddish-red	N	225	92.59	18	7.41	2	0.72	0.000	1.000
	Y	31	93.94	2	6.06				
Purple	N	193	96.02	8	3.98	12	4.35	11.741	<0.001^#^
	Y	63	84.00	12	16.00				
Blue	N	256	92.75	20	7.25	0	0.00		NA
	Y	0	0.00	0	0.00				
Bruise	N	251	92.62	20	7.38	0	0.00		1.000^a^
	Y	5	100.00	0	0.00				
Tongue shape									
Normal	N	147	89.10	18	10.90	2	0.72	8.189	0.004^**^
	Y	109	98.20	2	1.80				
Withered	N	256	92.75	20	7.25	0	0.00		NA
	Y	0	0.00	0	0.00				
Old	N	241	93.77	16	6.23	4	1.45	3.791	0.052
	Y	15	78.95	4	21.05				
Tender	N	251	92.62	20	7.38	0	0.00		1.000^a^
	Y	5	100.00	0	0.00				
Fat	N	223	95.30	11	4.70	9	3.26	12.440	<0.001^#^
	Y	33	78.57	9	21.43				
Thin	N	253	92.67	20	7.33	0	0.00		1.000^a^
	Y	3	100.00	0	0.00				
Punctured	N	256	92.75	20	7.25	0	0.00		NA
	Y	0	0.00	0	0.00				
Cracked	N	236	92.91	18	7.09	2	0.72	0.000	1.000
	Y	20	90.91	2	9.09				
Teeth marks	N	150	95.54	7	4.46	13	4.71	4.210	0.040^*^
	Y	106	89.08	13	10.92				
Color of tongue fur									
White	N	152	89.94	17	10.06	3	1.09	5.131	0.023^*^
	Y	104	97.20	3	2.80				
Yellow	N	106	96.36	4	3.64	16	5.80	3.546	0.060
	Y	150	90.36	16	9.64				
Gray	N	255	93.07	19	6.93	1	0.36		0.140^a^
	Y	1	50.00	1	50.00				
Black	N	255	93.07	19	6.93	1	0.36		0.14^a^
	Y	1	50.00	1	50.00				
Quality of tongue fur									
Scanty	N	151	88.82	19	11.18	1	0.36	10.172	<0.001^#^
	Y	105	99.06	1	0.94				
Thick	N	196	98.49	3	1.51	17	6.16	34.952	<0.001^#^
	Y	60	77.92	17	22.08				
Moist	N	255	92.73	20	7.27	0	0.00		1.000^a^
	Y	1	100.00	0	0.00				
Dry	N	216	93.51	15	6.49	5	1.81	0.607	0.436
	Y	40	88.89	5	11.11				
Slippery	N	221	86.70	34	13.30	4	1.45	4.896	0.027^*^
	Y	82	95.30	4	4.70				
Greasy	N	153	95.63	7	4.38	13	4.71	4.670	0.031^*^
	Y	103	88.79	13	11.21				
Rotten	N	253	93.01	19	6.99	1	0.36		0.216^a^
	Y	3	75.00	1	25.00				
Peeling	N	253	93.01	19	6.99	1	0.36		0.216^a^
	Y	3	75.00	1	25.00				
Like peeling	N	250	93.28	18	6.72	2	0.72		0.107^a^
	Y	6	75.00	2	25.00				

N, No; Y, Yes. ^a^Fisher’s exact probability method, ^*^P < 0.05, ^**^P < 0.01, ^#^*P* < 0.001. NA, a frequency of 0 and no chi-square test condition.

In the univariate analysis of testing information, indicators with a positive rate of <3% for severity (such as *P*T, INR, TT, APTT, and CKMB) were excluded. Finally, nine indicators, namely NE (*p* = 0.007), LY (*p* < 0.001), PLT (*p* < 0.001), IL-6 (*p* < 0.001), PCT (*p* < 0.001), D-D (*p* < 0.001), CK (*p* < 0.001), AST (*p* < 0.001), and LDH (*p* < 0.001), were included, as shown in Table 7. Among them, CRP, IL-6, FDP, and D-D are highly correlated. Previous studies (20, 21) support the greater significance of IL-6, while D-D has a higher positivity rate. A lower *p*-value was selected for inclusion.

**Table 7:** Univariate analysis of testing information.

Test	*n*	Classification	Non-severe group		Severe group		Positivity rate for severity		*χ^2^*	*P*
			*n*	%	*n*	%	*n*	%		
WBC	379	Normal	279	88.29	37	11.71	13	3.43	3.654	0.056
		Abnormal	50	79.37	13	20.63				
NE	379	Normal	266	89.26	32	10.74	18	4.75	7.334	0.007^**^
		Abnormal	63	77.78	18	22.22				
LY	379	Normal	255	94.44	15	5.56	35	9.23	47.812	<0.001^#^
		Abnormal	74	67.89	35	32.11				
HGB	379	Normal	235	88.35	31	11.65	19	5.01	1.844	0.174
		Abnormal	94	83.19	19	16.81				
PLT	364	Normal	282	90.68	29	9.32	14	3.85	12.696	<0.001^#^
		Abnormal	39	73.58	14	26.42				
CRP	371	Normal	199	99.00	2	1.00	48	12.94	58.609	<0.001^#^
		Abnormal	122	71.76	48	28.24				
IL-6	298	Normal	166	98.22	3	1.78	42	14.09	54.073	<0.001^#^
		Abnormal	87	67.44	42	32.56				
PCT	323	Normal	258	95.20	13	4.80	33	10.22	122.939	<0.001^#^
		Abnormal	19	36.54	33	63.46				
PT	316	Normal	269	86.77	41	13.23	3	0.95		0.037^*a^
		Abnormal	3	50.00	3	50.00				
INR	316	Normal	270	86.54	42	13.46	2	0.63		0.095^a^
		Abnormal	2	50.00	2	50.00				
APTT	315	Normal	253	88.15	34	11.85	10	3.17	10.189	0.001^**^
		Abnormal	18	64.29	10	35.71				
TT	315	Normal	265	86.32	42	13.68	2	0.63	0.156	0.693
		Abnormal	6	75.00	2	25.00				
Fib	315	Normal	153	91.07	15	8.93	29	9.21	7.609	0.006^**^
		Abnormal	118	80.27	29	19.73				
FDP	281	Normal	170	90.91	17	9.09	19	6.76	6.927	0.008^**^
		Abnormal	75	79.79	19	20.21				
D-D	313	Normal	201	92.63	16	7.37	29	9.27	28.192	<0.001^#^
		Abnormal	67	69.79	29	30.21				
PTA	316	Normal	264	86.84	40	13.16	4	1.27	2.418	0.12
		Abnormal	8	66.67	4	33.33				
CK	320	Normal	241	89.59	28	10.41	18	5.63	21.570	<0.001^#^
		Abnormal	33	64.71	18	35.29				
CKMB	295	Normal	245	86.57	38	13.43	6	2.03	9.422	0.002^**^
		Abnormal	6	50.00	6	50.00				
ALT	342	Normal	243	87.73	34	12.27	11	3.22	0.996	0.318
		Abnormal	54	83.08	11	16.92				
AST	349	Normal	238	91.89	21	8.11	28	8.02	29.283	<0.001^#^
		Abnormal	62	68.89	28	31.11				
Cr	345	Normal	236	88.06	32	11.94	15	4.35	2.890	0.089
		Abnormal	62	80.52	15	19.48				
LDH	320	Normal	169	93.89	11	6.11	36	11.25	24.151	<0.001^#^
		Abnormal	104	74.29	36	25.71				

^a^Fisher’s exact probability method, ^*^P < 0.05, ^**^P < 0.01, ^#^*P* < 0.001.

### 3.2 Binary unconditional stepwise logistic analysis

1 Warning indicators of severity without testing information.

Including positive indicators of general information (four items), clinical symptom (six items), and tongue feature (five items), with severity or not as an outcome, through binary unconditional stepwise logistic analysis, we obtained four correlations, namely age (*p* < 0.001), fever (*p* < 0.001), fat tongue shape (*p* < 0.001), and thick tongue fur (*p* < 0.001), as shown in Table 8.

2 Warning indicators of severity without tongue feature.

**Table 8:** Warning indicators of severity without testing information by logistic analysis.

Indicator	*β*	*S_1_*	*Wald*	*P*	*OR*	95% CI	
						Lower	Upper
Age (year)	0.098	0.021	22.282	<0.001^#^	1.104	1.059	1.150
Fever (yes)	3.340	0.594	31.616	<0.001^#^	28.209	8.807	90.356
Fat tongue shape (yes)	2.526	0.719	12.325	<0.001^#^	12.501	3.052	51.213
Thick tongue fur (yes)	2.569	0.774	11.005	<0.001^#^	13.050	2.861	59.529

^*^P < 0.05, ^**^P < 0.01, ^#^*P* < 0.001. *β*, partial regression coefficient; S_1_, standard error of *β*_1_; OR, odds ratio; 95% CI, 95% confidence interval.

Including positive indicators of general information (four items), clinical symptom (six items), and testing information (five items), with severity or not as an outcome, through binary unconditional stepwise logistic analysis, we obtained three correlations, namely age (*p* < 0.001), fever (*p* < 0.001), and PCT (*p* < 0.001), as shown in Table 9.

3 Aggregate indicators.

**Table 9:** Warning indicators of severity without tongue feature by logistic analysis.

Indicator	*β*	*S_1_*	*Wald*	*P*	*OR*	95% CI	
						Lower	Upper
Age (year)	0.105	0.020	27.345	<0.001^#^	1.111	1.068	1.156
Fever (yes)	2.701	0.638	17.905	<0.001^#^	14.890	4.262	52.020
PCT (>0.1 ng/mL)	2.812	0.568	24.474	<0.001^#^	16.640	5.462	50.694

^*^P < 0.05, ^**^P < 0.01, ^#^*P* < 0.001. *β*, partial regression coefficient; S_1_, standard error of *β*_1_; OR, odds ratio; 95% CI, 95% Confidence interval.

Including positive indicators of the above two parts, with severity or not as an outcome, through binary unconditional stepwise logistic analysis, we obtained five correlations, namely age (*p* < 0.001), fever (*p* < 0.001), PCT (*p* < 0.001), thick tongue fur (*p* = 0.004), and fat tongue shape (*p* = 0.002), as shown in Table 10.

4 Model comparison.

**Table 10:** Aggregate warning indicators of severity by logistic analysis.

Indicator	*β*	*S_1_*	*Wald*	*P*	*OR*	95% CI	
						Lower	Upper
Age (year)	0.103	0.022	21.456	<0.001^#^	1.109	1.061	1.158
Fever (yes)	3.146	0.648	23.565	<0.001^#^	23.232	6.524	82.728
PCT (>0.1 ng/mL)	2.491	0.564	19.498	<0.001^#^	12.077	3.997	36.491
Thick tongue fur (yes)	2.495	0.852	8.588	0.003^**^	12.127	2.285	64.356
Fat tongue shape (yes)	2.385	0.826	8.327	0.004^**^	10.858	2.149	54.854

^*^P < 0.05, ^**^P < 0.01, ^#^*P* < 0.001. *β*, partial regression coefficient; S_1,_ standard error of *β*_1_; OR, odds ratio; 95% CI, 95% Confidence interval.

We evaluated three logistic models using the Cox and Snell R and Negelkerke R methods. The closer the score is to 1, the better the fit. Aggregate indicators performed the best, as shown in Table 11.

**Table 11:** Comparison of three logistic models.

Model	Cox and Snell R	Negelkerke R
Model without testing information	0.341	0.650
Model without tongue feature	0.372	0.709
Aggregate model	0.388	0.741

## 4 Discussion

This study highlights the prognostic value of integrating traditional Chinese medicine (TCM) tongue features with clinical biomarkers. It has always been challenging to associate tongue features with systemic inflammation. Studies have found that increased reproductive activity of glossal epithelial cells is one of the main characteristics in the formation of thick, greasy tongue fur, and that increased vascular permeability is closely associated with its formation (22). COVID-19 is a virus that causes systemic inflammatory storms and a concomitant increase in vascular permeability. The oral cavity and intestine are the main distribution sites for human digestive bacteria. Some data suggest that the abundance of the same flora in both sites may follow a common change trend (23). Systemic immune dysfunction and bacteriological disorders, which are usually present in severe patients of COVID-19, may be related. Changes in tongue features are associated with systemic metabolism. A fat tongue is associated with abnormal upper airway patency and whole-body adiposity (24). This means that these patients have an underlying condition of airway problems and increased airway resistance and are also more likely to develop serious conditions, such as respiratory failure. These factors appear to be connected. Thick fur and fat shape reflect systemic inflammation and fluid retention in TCM theory, aligning with severe COVID-19 pathophysiology (e.g., cytokine storm, endothelial dysfunction, and airway problems).

According to Traditional Chinese Medicine theory, tongue diagnosis serves as a significant indicator for evaluating disease progression and severity. They help summarize the main etiologies of severe acute respiratory diseases from a TCM perspective (25). Studies have shown that the characteristics of the tongue and tongue coating in patients with severe acute respiratory syndrome are distinct, indicating a close relationship between the tongue appearance and the state of illness (26, 27). Other studies suggested that patients diagnosed with severe COVID-19 had a purple tongue and yellow tongue coating, while non-severe ones commonly had a light red tongue and white tongue coating. Tongue features can serve as potential indicators for evaluating disease prognosis (28). These studies suggest that while tongue features may predict disease progression, their relevance can vary over time, across locations, and with different strains.

This retrospective observational study may be subject to bias due to factors such as inaccurate data, patient memory errors, and different photo exposures. While we made every effort to verify the data, some bias may still exist. Moreover, different seasons and strains of the virus may produce variations in tongue features. This study was conducted during the summer amid the Delta variant pandemic.

## 5 Conclusion

Age, fever, PCT, thick tongue fur, and fat tongue shape together form a robust predictive model for COVID-19 severity. TCM-derived indicators contribute to early risk stratification, particularly in settings with limited laboratory access. Further validation across diverse populations and variants is warranted.

## Data Availability

The original contributions presented in the study are included in the article/Supplementary material, further inquiries can be directed to the corresponding authors.
