# Supplementary material for: Warning indicators of COVID-19 severity: a retrospective observational study integrating modern biomarkers and traditional tongue features
Source: Front Med (Lausanne). 2025 Apr 15;12:1500605. doi: 10.3389/fmed.2025.1500605 (PMC12037591; doi:10.3389/fmed.2025.1500605)
Supplement: Supplementary file 1 [file Table_1.docx]

Appendix 1: Abbreviations

| Abbreviation | Full title |
| --- | --- |
| ECMO | Extracorporeal Membrane Oxygenation |
| WBC | White blood cell |
| NE | Neutrophil |
| LY | Lymphocyte |
| HGB | Hemoglobin |
| PLT | Platelet |
| CRP | C-reactive protein |
| IL-6 | Interleukin 6 |
| PCT | Procalcitonin |
| APTT | Activated partial thromboplastin time |
| PT | Prothrombin time |
| TT | Thrombin time |
| Fib | Fibrinogen |
| FDP | Fibrinogen degradation products |
| D-D | D-Dimer |
| INR | International normalized ratio |
| PTA | Prothrombin activity |
| CK | Creatine kinase |
| CKMB | Creatine kinase isoenzyme |
| ALT | Glutamic-pyruvic transaminase |
| AST | Glutamic oxaloacetic transaminase |
| Cr | Creatinine |
| LDH | Lactic dehydrogenase |
| CI | Confidence interval |
| OR | Multivariate-adjusted odd ratio |
| TNF-α | Tumor necrosis factor-α |
| sCys C | Serum cystatin C |
| BMI | Body Mass Index |
| LPS | Lipopolysaccharides |
| ICU | Intensive Care Unit |
