# Supplementary material for: Warning indicators of COVID-19 severity: a retrospective observational study integrating modern biomarkers and traditional tongue features
Source: Front Med (Lausanne). 2025 Apr 15;12:1500605. doi: 10.3389/fmed.2025.1500605 (PMC12037591; doi:10.3389/fmed.2025.1500605)
Supplement: Supplementary file 2 [file Table_2.docx]

Appendix 2: Tongue feature example


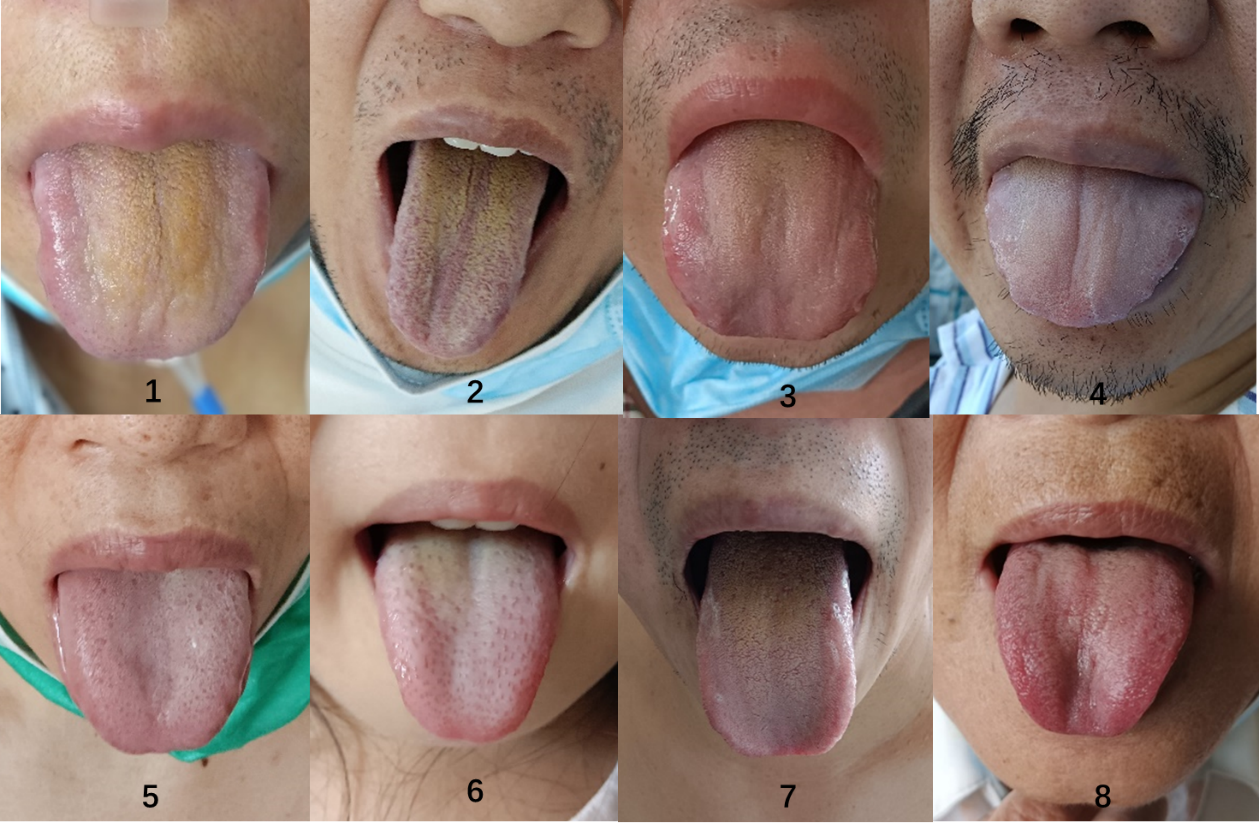


| NO. | Tongue color | Tongue shape | Color of tongue fur | Quality of tongue fur |
| --- | --- | --- | --- | --- |
| 1 | light red | fat | yellow | greasy |
| 2 | purple | thin | yellow | dry; thick |
| 3 | light red | fat | yellow | scanty |
| 4 | light red | fat; teeth marks | white | slippery |
| 5 | light red | fat | white | scanty |
| 6 | red | thin | scanty | greasy |
| 7 | red | thin | yellow | dry; thick |
| 8 | reddish-red | fat | white | Scanty |
