# Supplementary material for: Warning indicators of COVID-19 severity: a retrospective observational study integrating modern biomarkers and traditional tongue features
Source: Front Med (Lausanne). 2025 Apr 15;12:1500605. doi: 10.3389/fmed.2025.1500605 (PMC12037591; doi:10.3389/fmed.2025.1500605)
Supplement: Supplementary file 3 [file Table_3.docx]

**Appendix 3 Table of Normal Reference Ranges for Tests**

|  | Xiamen | | Guangzhou | |
| --- | --- | --- | --- | --- |
| test | unit | Normal Reference | unit | Normal Reference |
| WBC | 10^9^/L | 3.5-9.5 | 10^9^/L | 3.5-9.5 |
| NE | 10^9^/L | 1.8-6.3 | 10^9^/L | 1.8-6.3 |
| LY | 10^9^/L | 1.1-3.2 | 10^9^/L | 1.1-3.2 |
| HGB | G/L | 115-150 | G/L | 115-150 |
| PLT | 10^9^/L | 125-350 | 10^9^/L | 125-350 |
| CRP | ng/L | 0-7 | ng/L | 0-10 |
| IL-6 | Pg/ml | 0-7 | Pg/ml | 0-5.3 |
| PCT | ng/ml | 0-0.1 | ng/ml | 0-0.1 |
| APTT | sec | 20-40 | sec | 21-37 |
| PT | sec | 9-15 | sec | 9.2-15 |
| TT | sec | 13-20 | sec | 10-20 |
| Fib | g/L | 2-4 | g/L | 2-4 |
| FDP | mg/l | 0-5 | mg/l | 0-5 |
| D-D | mg/l | 0-0.55 | mg/l | 0-0.55 |
| INR | / | 0.73-1.28 | / | 0.8-1.25 |
| PTA | % | 70-130 | % | 70-130 |
| CK | U/L | 38-174 | U/L | 40-200 |
| CKMB | U/L | 0.1-24 | U/L | 0-24 |
| ALT | U/L | 7-40 | U/L | 7-40 |
| AST | U/L | 13-35 | U/L | 13-35 |
| Cr | umol/L | 53-97 | umol/L | 41-81 |
| LDH | U/L | 91-180 | U/L | 120-250 |

Note：Normal range including before and after thresholds.
