# Supplementary material for: Warning indicators of COVID-19 severity: a retrospective observational study integrating modern biomarkers and traditional tongue features
Source: Front Med (Lausanne). 2025 Apr 15;12:1500605. doi: 10.3389/fmed.2025.1500605 (PMC12037591; doi:10.3389/fmed.2025.1500605)
Supplement: Supplementary file 4 [file Table_4.docx]

**Appendix 4 Table of testing information**

| **Test** | **n** | **unit** | **Mean** | **Median** | **Quartiles** | | |
| --- | --- | --- | --- | --- | --- | --- | --- |
|  |  |  |  |  | **25** | **50** | **75** |
| **WBC** | 379 | 10^9^/L | 5.77 | 5.49 | 4.24 | 5.49 | 6.82 |
| **NE** | 379 | 10^9^/L | 3.50 | 3.16 | 2.17 | 3.16 | 4.23 |
| **LY** | 379 | 10^9^/L | 1.76 | 1.65 | 1.12 | 1.65 | 2.23 |
| **HGB** | 379 | G/L | 132.03 | 132.00 | 121.00 | 132.00 | 144.00 |
| **PLT** | 364 | 10^9^/L | 205.55 | 192.00 | 154.00 | 192.00 | 245.00 |
| **CRP** | 371 | ng/L | 25.80 | 10.00 | 2.61 | 10.00 | 30.10 |
| **IL-6** | 298 | Pg/ml | 34.10 | 3.36 | 1.50 | 3.36 | 25.57 |
| **PCT** | 323 | ng/ml | 0.16 | 0.05 | 0.05 | 0.05 | 0.06 |
| **PT** | 316 | sec | 11.81 | 11.60 | 11.10 | 11.60 | 12.45 |
| **INR** | 316 | sec | 1.05 | 1.04 | 1.00 | 1.04 | 1.09 |
| **APTT** | 315 | sec | 32.16 | 31.78 | 28.53 | 31.78 | 35.00 |
| **TT** | 315 | g/L | 16.92 | 16.70 | 16.00 | 16.70 | 17.64 |
| **Fib** | 315 | mg/l | 4.09 | 3.97 | 3.18 | 3.97 | 4.74 |
| **FDP** | 281 | mg/l | 4.38 | 4.00 | 2.80 | 4.00 | 5.22 |
| **D-D** | 313 | / | 0.63 | 0.41 | 0.25 | 0.41 | 0.61 |
| **PTA** | 316 | % | 92.24 | 92.00 | 83.09 | 92.00 | 101.60 |
| **CK** | 320 | U/L | 112.91 | 74.00 | 53.00 | 74.00 | 116.75 |
| **CKMB** | 295 | U/L | 10.82 | 10.00 | 6.00 | 10.00 | 14.00 |
| **ALT** | 342 | U/L | 29.08 | 21.00 | 15.15 | 21.00 | 34.00 |
| **AST** | 349 | U/L | 31.05 | 24.10 | 19.00 | 24.10 | 33.30 |
| **Cr** | 345 | umol/L | 67.60 | 64.90 | 55.00 | 64.90 | 78.60 |
| **LDH** | 320 | U/L | 280.43 | 271.00 | 218.00 | 271.00 | 336.75 |

Note: If the test result was CRP≤10, IL-6≤1.5, PCT≤0.05, it was recorded as 10, 1.5, 0.05.
